# Factors associated with subsequent fractures post-hospital discharge for hip fracture: A single institution, retrospective cohort study

**DOI:** 10.1016/j.jarlif.2026.100075

**Published:** 2026-06-04

**Authors:** Yuto Kasai, Ryo Kitsu, Takumi Miyoshi, Tsubasa Hochido, Ryoko Sakai, Manabu Akazawa

**Affiliations:** a Department of Public Health and Epidemiology, Meiji Pharmaceutical University, Tokyo, Japan; b Department of Pharmacy, Higashi-Kawaguchi Hospital, Saitama, Japan

**Keywords:** Hip fracture, Subsequent fracture, Osteoporosis, Fracture risk

## Abstract

Hip fractures in older adults are associated with substantial morbidity, loss of independence, and increased mortality, particularly in an aging society such as Japan. Although the risk of subsequent fractures after hip fracture is well recognized, discharge-related risk factors have not been fully evaluated. We conducted a retrospective cohort study using electronic medical records from Higashi-Kawaguchi Hospital, including patients hospitalized for hip fractures between April 1, 2015, and March 31, 2020. Patients were followed from the date of discharge to the date of subsequent fracture or to the last date of a confirmed medical record by our hospital until March 31, 2022. The incidence of subsequent fractures was determined, and hazard ratios (HRs) were calculated using Cox proportional hazards analysis to identify discharge-related risk factors.

Among the 620 patients included, 131 patients (21.1%) experienced subsequent fractures, with 60 (9.7%) occurring within 12 months after discharge. Patients with subsequent fractures were more likely to be female, significantly older, and showed differences in osteoporosis medication use and the use of fracture risk–associated drugs in unadjusted analyses. The Cox proportional hazards model identified age by decade (HR: 1.71; 95% CI 1.35–2.16) and lower body mass index (BMI) (HR: 0.92; 95% CI: 0.86–0.97) as significant risk factors for subsequent fractures.

These findings highlight the importance of discharge-based risk assessment and targeted post-discharge interventions for older patients and those with low BMI, given the high incidence of subsequent fractures within 12 months after discharge.

## 1 Introduction

Hip fractures are a major cause of morbidity in older people, leading to declines in physical function, loss of independence, and increased risk of mortality [1]. In aging populations such as Japan, hip fractures are prevalent and contribute to social problems such as increased medical care demands and healthcare costs [2,3]. In addition, hip fractures are reportedly associated with the risk of subsequent fractures, requiring further medical care and increased healthcare costs [3,4].

In Japan, patients with hip fractures are typically treated surgically during hospitalization, followed by a period of inpatient care and rehabilitation. Because the target goals for each patient differ depending on pre-fracture activities of daily living (ADL), in-hospital course, and discharge destination, multidisciplinary interventions are tailored to the individual patient. Post-discharge follow-up varies depending on the hospital and patient background, but is commonly provided through outpatient visits, home-visit nursing, or rehabilitation services. Although electronic medical records are widely used within individual institutions, comprehensive nationwide data linkage systems remain limited. Therefore, clinical information such as bone mineral density (BMD), fall risk, body mass index (BMI), ADL, place of residence, and discharge destination is often derived from data from individual institutions. These characteristics may differ from healthcare systems in other countries, where integrated data linkage and standardized post-discharge care pathways are more commonly established.

The incidence of subsequent fractures following hip fractures in Japan was reported to be 70 per 1000 person-years [4]. Older age, renal disease, dementia, and Parkinson’s disease have been identified as risk factors for subsequent hip fracture in patients with a prior hip fracture [5]. An observational study reported that the risk factors for subsequent vertebral fractures were history of vertebral fracture, medication possession rate (MPR) under 80% for osteoporosis treatment, and lower lumbar BMD [6]. Previous studies have assessed patient baseline status before or at the time of initial hip fracture or subsequent fractures. However, the characteristics of patients at discharge that may be associated with subsequent fractures remain unclear. Hip fractures have been reported to be associated with postoperative complications and a decline in motor function [1,7], with a different clinical condition expected at the time of discharge compared to the time of admission. Importantly, most previous studies have focused on patient characteristics at baseline or at the time of fracture. In contrast, the present study specifically focuses on clinical characteristics at the time of hospital discharge, reflecting a clinically relevant transition point when treatment decisions and follow-up planning are made. By using routinely available discharge variables, this study aimed to provide a practical approach to risk stratification that can be readily implemented in real-world hospital settings. To improve preventive strategies and prevent subsequent fractures after discharge in patients with hip fractures, risk factors identifiable at discharge should be evaluated.

We aimed to investigate the frequency of subsequent fractures in patients hospitalized for hip fractures and to identify discharge-related risk factors that may predict such events.

## 2 Methods

### 2.1 Study cohort and follow-up

This retrospective cohort study utilized electronic medical records from Higashi-Kawaguchi Hospital in Saitama Prefecture, Japan—a facility with 198 beds, including general wards, general disabled wards, and convalescent rehabilitation wards. Patients hospitalized for a hip fracture between April 1, 2015, and March 31, 2020, were screened. Patients were excluded if they were aged <50 years, died during hospitalization, were transferred to a different hospital, received only conservative therapy for hip fracture, had a hip fracture due to high-energy trauma, had no post-discharge records of orthopedic outpatient visits, home-visit nursing, home-visit rehabilitation, or outpatient rehabilitation. The study cohort was observed from the date of discharge to the date of a subsequent fracture or the last date of a confirmed visit to our hospital until March 31, 2022.

#### 2.1.1 Definition of subsequent fracture

Subsequent fracture was defined as any skeletal fracture in the post-discharge study cohort. The skeletal site of the fracture was classified as hip, vertebral, non-hip non-vertebral (NHNV) fracture, periprosthetic, or multiple fractures.

### 2.2 Covariates

Data on age, sex, BMI, the walk/wheelchair item of the functional independence measure (FIM), comorbidities, number of drugs, osteoporosis medications, and drugs with fracture risk were collected at the date of discharge or nearest date of discharge. In addition, changes in residence between admission and discharge destination were examined. The FIM was designed to assess the amount of assistance required for activities of daily living. It consists of a total of 18 items, including motor and cognitive domains, with each item scored on a 7-point scale ranging from 1 to 7. Higher scores indicate greater independence, with a score of 1 representing total assistance and a score of 7 representing complete independence. In this study, the walk/wheelchair item was assessed to evaluate walking status at discharge [8]. The locomotion (walk/wheelchair) item of the FIM was used as a measure of mobility and level of assistance at discharge. These factors are clinically important indicators of functional status and are closely associated with fall risk. In contrast, composite FIM scores include multiple domains, such as mobility, self-care, and cognitive function, which may not be equally or directly related to fall risk after hip fracture. Therefore, the use of a total score may obscure the specific contribution of mobility, which was the primary focus of this study and was considered more interpretable for clinical application at discharge. Comorbidities, including cognitive decline, cerebrovascular disease, Parkinson’s disease, epilepsy, chronic obstructive pulmonary disease, arrhythmia, angina, myocardial infarction, diabetes mellitus, hypertension, dyslipidemia, chronic kidney disease, thyroid disease, rheumatoid arthritis, knee osteoarthritis, gastrectomy, and visual disorders, have been reported as risk factors for fractures. Cognitive decline was defined as a diagnosis of dementia, fewer than 21 points on the revised version of Hasegawa’s dementia scale, or fewer than 24 points on the Mini-Mental State Examination. The number of drugs was categorized as polypharmacy (≧6) or non-polypharmacy (<6). Medications for osteoporosis included calcium, estrogen, active vitamin D3, bisphosphonates, selective estrogen receptor modulators, calcitonin, teriparatide, denosumab, ipriflavone, nandrolone decanoate, and vitamin K2. In addition, we evaluated the timing of initiation of osteoporosis medications and the presence or absence of prescriptions after hospital discharge, and calculated the MPR in patients prescribed osteoporosis treatments. Drugs with a fracture risk were defined as drugs with a reported risk of fracture, osteoporosis, falls, oversedation, delirium, cognitive decline, orthostatic hypotension, hypoglycemia, extrapyramidal symptoms, and Parkinson’s-like dystonic symptoms. These drugs were considered based on “List of drugs to be prescribed with special caution,” which is an extract from the “Guidelines for medical treatment and its safety in the elderly 2015” [9]. In addition, proton pump inhibitors (PPIs), which are reportedly associated with fracture risk, were included in this analysis [10].

### 2.3 Statistical analysis

Patient characteristics were recorded and compared between those with and without subsequent fractures using the *t*-test for continuous variables and the chi-square test or Fisher’s exact test for categorical variables, as appropriate. In patients with subsequent fractures, the period from the date of discharge to the date of subsequent fracture and the site of the fracture were recorded. Cox proportional hazards analysis was used to identify risk factors for subsequent fractures, and hazard ratios (HRs) with 95% confidence intervals (95% CIs) were calculated. Covariates included in the multivariate analysis were age, sex, BMI, number of comorbidities (0, 1, or >1), fracture history, use of osteoporosis medicines, use of drugs with fracture risk, the locomotion (walk/wheelchair) item of the FIM, and a change in discharge destination. The proportional hazards assumption was assessed using Schoenfeld residuals, and a history of fracture was found to violate this assumption; therefore, history of fracture was included as a stratification variable in a stratified Cox regression analysis. This approach allows the baseline hazard to differ by fracture history, while assuming proportional effects of other covariates within each stratum. In addition, to provide a more intuitive understanding of the association between subsequent fracture and identified risk factors, we described Kaplan–Meier curves stratified by risk factors at discharge. Two-tailed statistical significance was set at p < 0.05. R Studio (version 4.3.1) was used for statistical analyses.

### 2.4 Ethics approval and consent to participate

This study was approved by the Ethics Review Committee of Higashi-Kawaguchi Hospital (April 2022) and Meiji Pharmaceutical University (approval number 202,227).

## 3 Results

### 3.1 Patient characteristics

A total of 1325 patients were hospitalized for hip fractures between April 1, 2015, and March 31, 2020. After applying the exclusion criteria, 620 patients were included in this study. The mean follow-up duration was 12.4 ± 15.36 months, with a median of 5 months, ranging from 0 to 73 months. Patient characteristics are shown in Table 1. The mean age was 81.7 years and 501 patients were female (80.8%). Patients had a mean BMI of 19.76 ± 3.18, and a median FIM (walk/wheelchair) score was 5.0 [3.0, 6.0]. At discharge, 117 (18.8%) patients were prescribed osteoporosis medications, 399 (64.3%) received drugs associated with fracture risk, and 288 (46.5%) were prescribed ≥6 medications. A total of 131 patients (21.1%) were diagnosed with subsequent fractures after discharge. Compared to those without subsequent fractures, patients with subsequent fractures showed significant differences in age, female sex, osteoporosis medication use, and use of drugs associated with fracture risk. No significant differences were observed for other factors.

**Table1:** Patient characteristics.

	Overall (n = 620)	Without subsequent fracture (n = 489)	Subsequent fracture (n = 131)	P value
Age	81.66 (8.97)	81.13 (9.27)	83.63 (7.45)	0.005
Age by decade				0.077
50–59	14 (2.3)	14 (2.9)	0 (0.0)	
60–69	43 (6.9)	39 (8.0)	4 (3.1)	
70–79	167 (26.9)	134 (27.4)	33 (25.2)	
80–89	275 (44.4)	213 (43.6)	62 (47.3)	
90–99	117 (18.9)	86 (17.6)	31 (23.7)	
≥100	4 (0.6)	3 (0.6)	1 (0.8)	
Female	501 (80.8)	385 (78.7)	116 (88.5)	0.016
BMI	19.76 (3.18)	19.85 (3.25)	19.40 (2.87)	0.154
<18.5	243 (39.2)	190 (38.9)	53 (40.5)	0.816
18.5–25	335 (54.0)	262 (53.6)	73 (55.7)	0.735
≥25	42 (6.8)	37 (7.6)	5 (3.8)	0.187
FIM (walk/wheelchair)	5.00 [3.00, 6.00]	5.00 [2.00, 6.00]	5.00 [4.00, 6.00]	0.161
History of fracture^a^	144 (23.2)	112 (22.9)	32 (24.4)	0.802
Number of comorbidity				
0	66 (10.6)	55 (11.2)	11 (8.4)	0.435
1	93 (15.0)	78 (16.0)	15 (11.5)	0.253
≥2	448 (72.3)	346 (70.8)	102 (77.9)	0.133
**Comorbidity**				
Cognitive decline	295 (47.6)	233 (47.6)	62 (47.3)	1
Cerebral vascular disorder	121 (19.5)	94 (19.2)	27 (20.6)	0.817
Epilepsy	8 (1.3)	6 (1.2)	2 (1.5)	0.678
Thyroid disease	20 (3.2)	16 (3.3)	4 (3.1)	1
Arrhythmia	57 (9.2)	43 (8.8)	14 (10.7)	0.62
Angina or MI	39 (6.3)	31 (6.3)	8 (6.1)	1
COPD	12 (1.9)	9 (1.8)	3 (2.3)	0.724
Diabetes mellitus	133 (21.5)	103 (21.1)	30 (22.9)	0.738
Hypertension	409 (66.0)	319 (65.2)	90 (68.7)	0.522
Hyperlipidemia	158 (25.5)	123 (25.2)	35 (26.7)	0.801
Rheumatoid arthritis	18 (2.9)	15 (3.1)	3 (2.3)	0.777
Knee osteoarthritis	20 (3.2)	9 (1.8)	11 (8.4)	0.00075
Renal disease	33 (5.3)	26 (5.3)	7 (5.3)	1
Eye disorder	141 (22.7)	106 (21.7)	35 (26.7)	0.269
Gastrectomy	18 (2.9)	14 (2.9)	4 (3.1)	1
Parkinson disease	28 (4.5)	20 (4.1)	8 (6.1)	0.453
**Medication**				
Osteoporosis medications	117 (18.9)	81 (16.6)	36 (27.5)	0.007
Bisphosphonate	58 (9.4)	35 (7.2)	23 (17.6)	0.00054
SERM	14 (2.3)	10 (2.0)	4 (3.1)	0.508
Vitamin D3	69 (11.1)	50 (10.2)	19 (14.5)	0.22
Teriparatide	1 (0.2)	1 (0.2)	0 (0.0)	1
Calcium	8 (1.3)	8 (1.6)	0 (0.0)	0.214
Other	4 (0.6)	3 (0.6)	1 (0.8)	1
Drugs with a fracture risk^b^	398 (64.2)	300 (61.3)	98 (74.8)	0.006
Typical antipsychotic	28 (4.5)	22 (4.5)	6 (4.6)	1.000
Atypical antipsychotic	35 (5.6)	28 (5.7)	7 (5.3)	1.000
Benzodiazepine	113 (18.2)	82 (16.8)	31 (23.7)	0.091
Non-benzodiazepine	65 (10.5)	53 (10.8)	12 (9.2)	0.692
Tricyclic antidepressant	8 (1.3)	4 (0.8)	4 (3.1)	0.115
SSRI	14 (2.3)	9 (1.8)	5 (3.8)	0.307
α1-receptor blockers (nonselective for receptor subtypes)	30 (4.8)	19 (3.9)	11 (8.4)	0.056
First-generation H1 receptor antagonists	3 (0.5)	3 (0.6)	0 (0.0)	0.849
Histamine H2 receptor antagonists	48 (7.7)	31 (6.3)	17 (13.0)	0.019
Loop diuretics	68 (11.0)	49 (10.0)	19 (14.5)	0.193
Anti-Parkinson’s drugs (anticholinergic drugs)	4 (0.6)	4 (0.8)	0 (0.0)	0.671
Antiemetic drugs	1 (0.2)	1 (0.2)	0 (0.0)	1.000
Sulpiride	7 (1.1)	6 (1.2)	1 (0.8)	1.000
Corticosteroids	18 (2.9)	12 (2.5)	6 (4.6)	0.320
Sulfonylureas	40 (6.5)	31 (6.3)	9 (6.9)	0.985
Biguanides	35 (5.6)	27 (5.5)	8 (6.1)	0.964
Thiazolidine derivatives	12 (1.9)	9 (1.8)	3 (2.3)	1.000
SGLT2 inhibiter	3 (0.5)	2 (0.4)	1 (0.8)	1.000
Insulin	19 (3.1)	16 (3.3)	3 (2.3)	0.769
PPI	180 (29.0)	144 (29.4)	36 (27.5)	0.740
Oxybutynin (oral)	0 (0.0)	0 (0.0)	0 (0.0)	1.000
Muscarinic receptor antagonists	23 (3.7)	13 (2.7)	10 (7.6)	0.016
Number of medicines ≥6	288 (46.5)	220 (45.0)	68 (51.9)	0.19
Change in discharge destination	85 (13.7)	70 (14.3)	15 (11.5)	0.482

Values are presented as mean (standard deviation) for continuous variables and count (percentage) for categorical variables, except for FIM (walk/wheelchair), which is presented as median (interquartile range).

BMI, body mass index; FIM, Functional Independence Measure; MI, myocardial infarction; COPD, chronic obstructive pulmonary disease; SERM, selective estrogen receptor modulator; SSRI, selective serotonin reuptake inhibitor; SGLT2, sodium-glucose cotransporter 2; PPI, proton pump inhibitor.

^a^History of a fracture prior to the hip fracture.

^b^Drugs with a fracture risk including medications for osteoporosis, falls, oversedation, delirium, cognitive decline, orthostatic hypotension, hypoglycemia, extrapyramidal symptoms, and Parkinson’s-like dystonic symptom were extracted from "List of drugs to be prescribed with special caution".

### 3.2 Patient characteristics with subsequent fracture

Among the study cohort, 131 (21.1%) had a subsequent fracture and 60 (9.7%) had a subsequent fracture within 12 months. Of these, 38 had hip fractures, 15 had vertebral fractures, 63 had NHNV fractures, eight had periprosthetic fractures, three had multiple fractures, and four had fractures at an unknown site.

### 3.3 Risk factors for subsequent fracture

HRs were calculated using Cox proportional hazards analysis to identify risk factors for subsequent fractures (Table 2). Age by decade (HR, 1.71; 95% CI, 1.35–2.16), and lower BMI (HR, 0.92; 95% CI, 0.86–0.97) were identified as significant risk factors for subsequent fracture.

**Table 2:** Cox proportional hazards analysis of risk factors for subsequent fracture post-hip fracture.

	HR (95% CI)
Age by decade	1.71 (1.35–2.16)
Female	1.45 (0.83–2.52)
BMI	0.92 (0.86–0.97)
Number of comorbidities	
0	Reference
1	0.73 (0.21–2.56)
≥2	1.14 (0.35–3.71)
Osteoporosis medications	1.47 (0.97–2.24)
Drugs with a fracture risk^a^	1.26 (0.83–1.90)
FIM (walk/wheelchair)	0.98 (0.88–1.10)
Change in discharge destination	1.52 (0.95–2.47)

HR, hazard ratio; 95% CI, 95% confidence interval; BMI, body mass index; FIM, Functional Independence Measure.

The model was adjusted for all variables listed in the table. History of fracture was used as a stratification factor and was therefore not included as a covariate. BMI was analyzed as a continuous variable (per 1 kg/m² increase). FIM indicates the locomotion item (walking/wheelchair) of the Functional Independence Measure and was analyzed per 1-point increase.

a Drugs with a fracture risk, including medications associated with osteoporosis, falls, oversedation, delirium, cognitive decline, orthostatic hypotension, hypoglycemia, extrapyramidal symptoms, and Parkinsonian or dystonic symptoms, were extracted from the “List of drugs to be prescribed with special caution”.

**Fig. 1:**
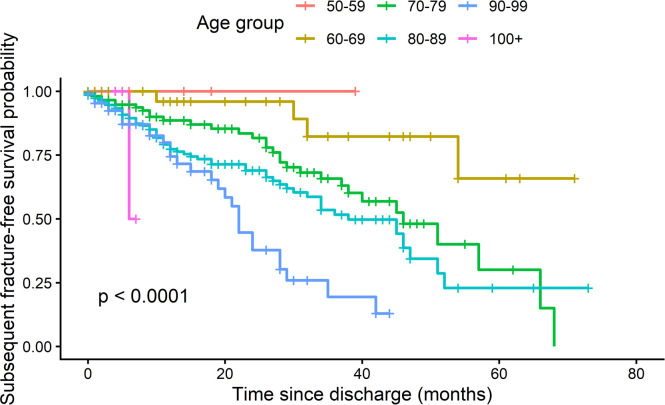
Kaplan–Meier curves for subsequent fracture-free survival stratified by age group here.

**Fig. 2:**
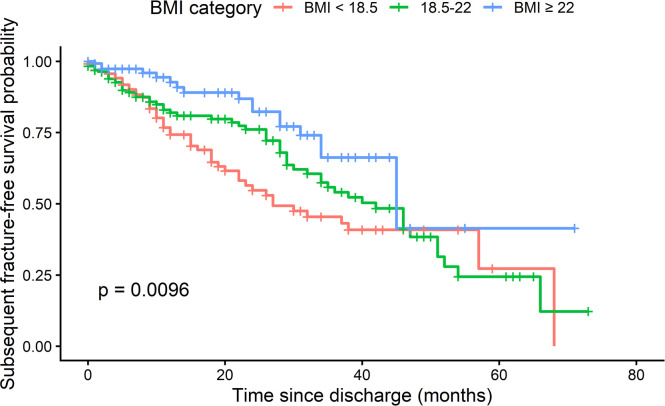
Kaplan–Meier curves for subsequent fracture-free survival stratified by BMI category at discharge here.

Fig. 1, Fig. 2 show Kaplan–Meier curves stratified by age group and BMI category at discharge. Patients with lower BMI showed a significantly higher risk of subsequent fracture compared with those with higher BMI (log-rank test, p = 0.0096). Similarly, a clear gradient of increasing fracture risk was observed with advancing age (log-rank test, p < 0.0001).

## 4 Discussion

This study identified older age and lower BMI as significant risk factors for subsequent fractures, which are consistent with previous studies. An important distinction of the present study lies in its focus on discharge-time assessment. Many prior studies have relied on baseline characteristics or at the time of fracture, which may not capture the clinical status of patients after in-hospital treatment and rehabilitation. In contrast, our study evaluates patients at the time of discharge, a clinically actionable time point when care transitions occur and preventive strategies can be initiated. Furthermore, by incorporating routinely available variables such as BMI, medication profile, and a simple functional measure (FIM locomotion), our findings highlight a pragmatic approach to risk stratification that can be applied in everyday clinical practice, particularly in settings without access to comprehensive registries or detailed frailty assessments.

The 12-month incidence of subsequent fractures (9.7%) was comparable to previous reports. A multicenter observational study in Japan reported a 6.6% incidence during the first year of follow-up [4], while a large population-based study reported an incidence of 8.4% for subsequent non-hip fractures[11]. Although the definition of a subsequent fracture in that study was different from ours, the percentage of patients with subsequent non-hip fractures was similar. The slightly higher incidence observed in our study may be partly explained by differences in patient selection, particularly the exclusion of patients who were transferred or lost to follow-up, which may have resulted in a cohort with relatively lower mortality risk and longer observation time. In addition, unlike several previous studies that focused on specific fracture types or used administrative data, our study provides a detailed characterization of subsequent fracture sites and timing within a single-institution clinical cohort, allowing for a more granular understanding of early post-discharge fracture patterns.

In this study, older age and lower BMI were identified as significant risk factors for subsequent fractures, consistent with previous reports [5,12,13]. In the multivariable analysis, both age (per 10-year increase) and BMI (per 1 kg/m² increase) were associated with subsequent fracture risk, with hazard ratios of 1.71 and 0.92, respectively, indicating a consistent gradient of risk. These factors likely reflect underlying physiological vulnerability, including declines in BMD, muscle mass, and balance, which collectively increase susceptibility to falls and fractures [13,14]. Although these factors are not easily modifiable, they are clinically useful for risk stratification at the time of discharge. In particular, low BMI may reflect underlying malnutrition and sarcopenia, which are potentially modifiable through nutritional and rehabilitative interventions.

From a clinical perspective, our findings suggest that risk stratification at the time of discharge may be a practical approach to identifying patients at high risk of subsequent fractures. In particular, older patients with low BMI may benefit from structured, multidisciplinary post-discharge interventions. These may include early initiation and optimization of osteoporosis treatment, targeted nutritional support to address low body weight, and individualized fall prevention strategies. In addition, the integration of these risk factors into discharge planning protocols or fracture liaison services may help ensure continuity of care and improve long-term outcomes. Given the high incidence of fractures within the first year, timely intervention immediately after discharge may be especially critical.

The association between medication use and subsequent fracture risk warrants careful interpretation. Although drugs associated with fracture risk showed a non-significant trend toward increased risk, this finding suggests that medication review may remain an important component of post-discharge care. Similarly, the observed trend toward increased fracture risk among patients receiving osteoporosis medications is likely explained by confounding by indication, as these patients may have had more severe underlying osteoporosis. Because BMD was not assessed in this study, residual confounding could not be fully addressed. In our cohort, the median FIM locomotion score at discharge was 5, corresponding to supervision or setup assistance, indicating that patients were generally able to ambulate or use a wheelchair independently but still required supervision or setup assistance for safety. Moreover, the FIM locomotion score at discharge was not significantly associated with subsequent fractures. This may be partly explained by the relatively limited variability in locomotion scores, with many patients clustering around moderate levels of independence at the time of discharge. In addition, as a single-item measure, the FIM locomotion score may not fully capture important dimensions of physical function and frailty, such as muscle strength, balance, and gait stability. It is also possible that the effects of functional status are partially mediated or confounded by age and BMI, which showed stronger associations in our model.

Importantly, a key strength of our findings is their feasibility in routine clinical settings. The identified risk factors—older age and lower BMI—are simple, routinely collected variables available at discharge, suggesting that even in busy hospital environments or in settings without access to BMD, detailed frailty assessments, or integrated registries, clinicians may be able to identify high-risk patients and prioritize follow-up care. These findings can be directly linked to feasible clinical actions, such as early referral to osteoporosis clinics, targeted nutritional interventions, and structured fall risk assessments prior to discharge. Although the effectiveness of these interventions was not evaluated, our results support the potential value of incorporating simple risk stratification into discharge planning. Furthermore, in resource-limited settings, these findings may help prioritize patients for enrollment in fracture liaison services or similar coordinated care programs, thereby optimizing the allocation of limited healthcare resources.

This study had some limitations. First, this was a single-institution study, and the findings may reflect specific care pathways and discharge practices unique to our setting, potentially limiting generalizability to other healthcare systems or settings. In addition, the exclusion of patients who were transferred or lost to follow-up may have introduced selection bias toward relatively healthier individuals, which could lead to an underestimation of mortality risk and an overestimation of subsequent fracture incidence. Second, the median follow-up duration was relatively short (5 months), although the range was wide. This may have resulted in a greater capture of early post-discharge fractures, while later fractures could have been missed, particularly if patients sought care at other institutions. Therefore, the long-term incidence of subsequent fractures may have been underestimated. Third, we could not evaluate fall risk and severity of osteoporosis because systematically gathered data on fall history and BMD were not available. This limitation may have resulted in residual confounding, particularly affecting the interpretation of medication-related variables. For example, patients receiving osteoporosis treatment may have had more severe underlying osteoporosis, potentially leading to an overestimation of the observed association. In addition, because fracture-risk–associated medications include agents that may induce osteoporosis and increase fall risk, the lack of assessment of BMD and fall risk may have further contributed to residual confounding, potentially resulting in an overestimation of their association with fracture risk. Fourth, although we included the locomotion (walk/wheelchair) item of the FIM as a proxy for functional status at discharge, this single-item measure may not fully capture important dimensions of frailty, such as muscle strength, gait speed, balance, and overall physical performance. In addition, we were unable to include more detailed frailty-related measures, such as gait speed, grip strength, or a formal frailty index, which are known to be associated with fracture risk and may interact with or mediate the effects of BMI and age. Therefore, the observed association between lower BMI and higher fracture risk may, in part, reflect underlying frailty or sarcopenia, which could not be directly assessed in this study. Because the study was not designed to comprehensively assess frailty, the roles of sarcopenia and functional decline as potential mediators remain inferential and require further investigation. Finally, although patient demographics and clinical characteristics may have changed post-discharge, we did not reassess them because post-discharge data were not available.

## 5 Conclusion

This study highlights the importance of discharge-based risk assessment and targeted post-discharge interventions for older patients and those with low BMI, given the high incidence of subsequent fractures within 12 months after discharge.

## Declarations

This study is an observational study and does not contain any identifiable personal data; therefore, informed consent for publication was not required.

## Availability of data and materials

The electronic health record data from Higashi-Kawaguchi Hospital used in this study have been anonymized to protect patient privacy, and the data are not shared with third parties.

## Consent for publication

Not applicable.

## Funding

The authors received no financial support for the study, authorship, or publication of this article.

## Declaration of generative AI and AI-assisted technologies in the writing process

During the preparation of this work, the author(s) used ChatGPT (OpenAI) in order to improve the clarity and readability of the English language. After using this tool, the author(s) reviewed and edited the content as needed and take(s) full responsibility for the content of the published article.

## CRediT authorship contribution statement

**Yuto Kasai:** Writing – review & editing, Writing – original draft, Project administration, Methodology, Investigation, Formal analysis, Conceptualization. **Ryo Kitsu:** Writing – review & editing, Methodology, Investigation, Conceptualization. **Takumi Miyoshi:** Writing – review & editing, Methodology, Investigation, Formal analysis. **Tsubasa Hochido:** Writing – review & editing, Methodology, Investigation, Data curation. **Ryoko Sakai:** Writing – review & editing, Supervision, Project administration, Conceptualization. **Manabu Akazawa:** Writing – review & editing, Supervision, Resources, Project administration, Methodology, Funding acquisition, Conceptualization.

## Declaration of competing interests

The authors declare the following financial interests/personal relationships which may be considered as potential competing interests:

Manabu Akazawa reports a relationship with Astellas Pharma Inc that includes: consulting or advisory. Manabu Akazawa reports a relationship with Janssen Pharmaceutical KK that includes: consulting or advisory. Manabu Akazawa reports a relationship with Kyowa Kirin Co., Ltd that includes: consulting or advisory. Manabu Akazawa reports a relationship with Mitsubishi Tanabe Pharma Corporation that includes: consulting or advisory. Manabu Akazawa reports a relationship with MSD KK that includes: consulting or advisory. Ryoko Sakai reports a relationship with Nippon Kayaku Co Ltd that includes: consulting or advisory. If there are other authors, they declare that they have no known competing financial interests or personal relationships that could have appeared to influence the work reported in this paper.
